# Gingival Crevicular Fluid (GCF): A Diagnostic Tool for the Detection of Periodontal Health and Diseases

**DOI:** 10.3390/molecules26051208

**Published:** 2021-02-24

**Authors:** Tauqeer Bibi, Zohaib Khurshid, Ambreen Rehman, Eisha Imran, Kumar Chandan Srivastava, Deepti Shrivastava

**Affiliations:** 1 Department of Orthodontics, Bahria University Medical and Dental College, Karachi 75260, Pakistan; tauqeerefatima@gmail.com; 2 Department of Prosthodontics and Dental Implantology, College of Dentistry, King Faisal University, Al-Ahsa 31982, Saudi Arabia; 3 Centre for Oral Immunobiology and Regenerative Medicine, Institute of Dentistry, Barts and The London, School of Medicine and Dentistry, Queen Mary University of London, London E1 2AT, UK; drambreenrehman@gmail.com; 4 Department of Dental Materials, HITEC Dental College, Institute of Medical Sciences, Taxilla 751010, Pakistan; eishaimran.dental@hitec-ims.edu.pk; 5 Oral Medicine and Radiology, Department of Oral Maxillofacial Surgery & Diagnostic Sciences, College of Dentistry, Jouf University, Sakaka 72345, Saudi Arabia; drkcs.omr@gmail.com; 6 Periodontics, Department of Preventive Dentistry, College of Dentistry, Jouf University, Sakaka 72345, Saudi Arabia

**Keywords:** periodontitis, inflammatory mediators, gingival crevicular fluid, proteomes, biomarkers, gingival inflammation

## Abstract

The methodologies applicable for the evaluation of periodontal associated diseases are constantly evolving to provide quick, realistic, and scientifically proven results. Trends in the past followed a clinical evaluation of periodontal tissues and radiographic-based reports that formed the foundation for detection of diseases involving the structures supporting the teeth. As the confines and limitations of conventional strategies became obvious over the passage of time, hand in hand variety of techniques have evolved and experimentally justified. These improvisations are based on an improved understanding of the periodontal-pathogenic cascade. Periodontal pathogenesis and a paradigm shift from disease understanding to disease prevention and treatment entail few prerequisites that demand the objectivity of diagnostics procedure that includes sensitivity and specificity along with an explanation of the intensity of the disease, Gingival crevicular fluid an oral bio-fluid resides in the close proximity with gingival tissues have been widely used to understand and differentiate the periodontal health and diseased status. The biomarkers present in the GCF can be a reliable tool to detect the minute changes seen in the disease processes. The GCF consists of various host and bacterial-derived products as well as biomarkers which in turn can be evaluated for the diagnosis, prognosis as well as management of the periodontal disease. Thus, the review aims at describing GCF as a potential oral biofluid helpful in differentiating periodontal health and disease status.

## 1. Introduction

Periodontal diseases have affected almost all the fields of dentistry in one way or the other. Foremost, every individual is affected differently by the periodontal disease. Besides, due to the inability to access rear-end segments of the oral cavity, clinical signs are sometimes inappreciable and the periodontal structures in the maxillary tuberosity region are difficult to visualize and properly examine. Additionally, clinical researches conducted in early times (approximately in the 1980s) showed that chronic periodontitis followed a slow and steady pattern of progression but later it became clear that clinical parameters are poor indicators of inactive states of disease pattern. However, Goodson in 1982 explained that it’s a natural tendency of chronic periodontitis showing regular pattern of occurrence which is interrupted by a phase of remission and acute exacerbation [1]. Day to day errors in the process of diagnosis and failure of treatment with the persistence of disease convinced investigators to devise a standard method for detection of disease. Presence and mapping the authentic treatment plan where the use of antibiotic is strictly administered in accordance to treat the symptoms.

In the light of multiple investigations performed, aiming at the detection of periodontal disease with absolute certainty, Table 1 provides a comprehensive view of the approaches that can help to achieve precision during the procedure. The secondary colonizing bacteria’s in the biofilm and the host-immune response plays a significant role in the commencement and its subsequent progression of periodontal disease. Although, the cascade of damage begins with alteration in normal microflora of the oral cavity, especially polymicrobial colonization in the form of biofilms on the tooth surface and derangements in subgingival flora. Over the time, the condition worsens due to power-play of consistent poor oral hygiene or any uncontrolled co-morbid condition and it presents as bad breath, bleeding from gums, pain, sensitivity and on occasion mobility of teeth. Physical assessment of the aforementioned symptoms and signs are the primary diagnostic criteria. However, clinical assessment alone cannot judge the status of the disease activity of the site.

Progressive destruction of collagen fibers that were initially limited to epithelium tissue advances to deeper supportive tissues of periodontium, including bone tissue that prevail state of periodontitis. Epidemiological studies have validated that the morbidity of chronic periodontitis is 35–50% in the adult population, with approximately 10% displaying severe disease concomitant with early tooth loss [2]. Whereas, this disease has increased the global burden by 57.3% from 1990 to 2010 [3]. It is an established fact that periodontitis has a polymicrobial etiology. It is not only the oral dysbiosis results in tissue destruction but also the host inflammatory dysregulation plays an important role in tissue destruction of periodontal supporting tissues [4]. The interesting fact is that components of host defense and pro-inflammatory mediator elements are raised during any periodontitis infection and infused locally as well as in gingival crevicular fluid (GCF) and saliva. Hence, oral liquids play a vital role in developing an accurate picture of periodontal health for obtaining data that mimics periodontium [5]. Zhou et al. conducted a study to find the microbiome and cytokine composition in GCF in healthy and periodontitis patients and they concluded that the oral dysbiosis and immune response plays a pivotal role in the etiology of periodontitis [6]. Similarly, Pei et al. collected the GCF from healthy and periodontitis patients and concluded that oral dysbiosis and changes in the metabolic product detected within GCF can be a good predictor of diagnosis, prognosis and management of periodontal disease [7]. Like other oral fluids, GCF is a reliable tool but unlike others, it is a very delicate specimen that maintains the structural integrity of the junctional epithelium (JE), hence acting as a barrier [8]. It is a serum transudate or inflammatory exudate fluid, present in the sulcus/periodontal pocket between the tooth and marginal gingiva. Talking about oral fluids, GCF is amongst the most localized fluid that delineates the very peculiar characteristics of the site, thus making future diagnostic treatment and management of a wide variety of maladies way easier [9]. Thus, considering the pivotal role of GCF being in the close proximity with the gingival tissues, this review paper aims to highlight the various aspects of GCF and its diagnostic potential for the discrimination of periodontal health and disease condition.

## 2. Gingival Crevicular Fluid

### 2.1. Formation of GCF

Early research in the field aimed to establish the nature of GCF; whether it is a transudate or exudate and its detection in the healthy gingiva. Researchers by the late 1950s and early 1960s had postulated that GCF is a result of stimulation by chemical or mechanical means which in turn results alters the permeability of the vessels beneath the junctional and sulcular epithelium [10]. Alfano and Pashley have reported that the initial fluid produced is interstitial and is produced in the crevice because of the osmotic gradient. Thus, the earlier was contemplated to be a transudate and later upon stimulation, it becomes inflammatory exudates [8].

### 2.2. Constituents of GCF Aliquot

**Figure 1:**
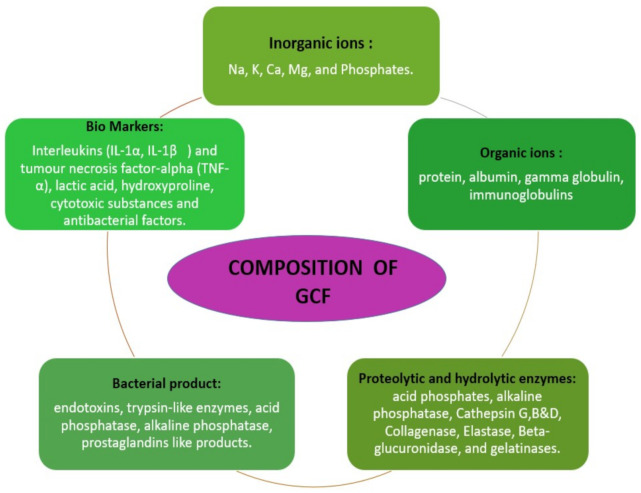
Description of gingival crevicular fluid composition [8].

The GCF is derived from serum and locally generated components such as tissue breakdown products and derivatives of subgingival biofilm. The various elements found in GCF includes inflammatory mediators, cytokines, leucocytes, enzymes, organic ions, tissue breakdown products and proteins. These components give an insight about the healing potential of periodontium. Conversely, it also reflects about the adaptive mechanism of certain bacterial survival within the gingival crevice and pocket [11]. The various biological disease markers such as interleukins (IL-Iα-IL-1β) Tumor necrosis factor alpha TNF α, enzymes such as acid phosphatase, alkaline phosphatase, matrix metalloproteinases, collagenases, elastase are widely used in periodontal research to assess the resolution of periodontal disease [12]. Along with this it also constitutes organic and inorganic ions (Figure 1).

### 2.3. Mechanism of Production

**Figure 2:**
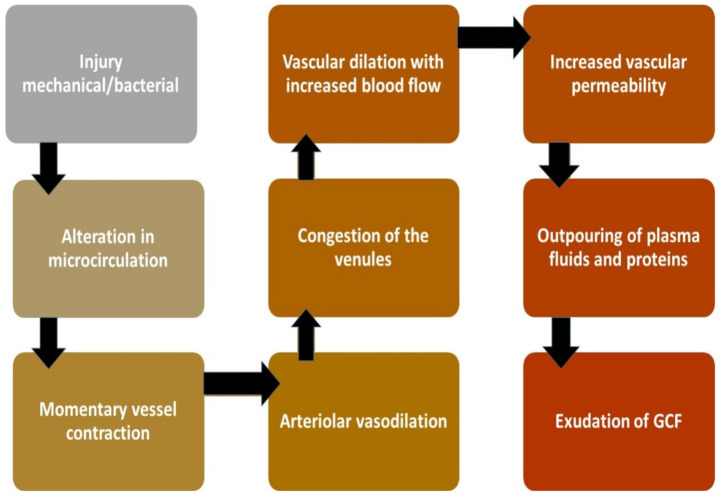
Mechanism of GCF production on stimulus [8,9,10].

Under normal conditions, the sulcus contains a minimal amount of GCF. During inflammation of tissue structure investing the tooth, this fluid in the V-shaped sulcus travels from the microcapillary framework within the inflamed connective tissues consisting of biological molecular markers. Hence, GCF is considered an important tool for assessing the health and diseased condition. In a healthy state, the sulcus of each tooth is flushed by GCF to get rid of the pathogens and toxic metabolites from periodontium and cushions the tooth against any foreign insult. However, during inflammation, it changes its properties from serum transudate to an inflammatory exudate where biological biomarkers including host defense molecules and anti-inflammatory mediators are present in enormous quantity hence the nature of the fluid is converted into exudates [8] (Figure 2).

### 2.4. Circadian Rhythm of GCF

Circadian periodicity of GCF is upsurged around early morning (6 a.m.) till night (10 p.m.) and down surged during the rest hours [13]. Other conditions like any changes in hormonal level, mechanical stimulations, smoking and periodontal therapy but particularly in case of a breach in the form of periodontal infection, activates host immune system to release polymorphonuclear leukocytes (PMNLs) and lymphocytes in GCF [13]. Other enzymes like proteinases are present already in the environment in its inactive proenzyme form and convert into its active form upon the stimulus of antigen by lymphocytes [14].

### 2.5. Collection of GCF

Collection of GCF is an easy but sensitive method. GCF collection can be achieved by employing any of the several proposed techniques [15] which can be chosen according to the aim or objective of the study. In the gingival washing method, the gingival crevice is flushed with an isotonic solution of fixed volume and then the diluted crevicular fluid is collected that contains both cells and soluble proteins [16]. This is a highly useful technique when the intent is to harvest cells from the gingival crevice region [17].

However, the properties of GCF such as composition or volume from specific sites cannot be assessed correctly as the dilution factor cannot be determined precisely [15]. Nonetheless, the GCF volume can be accurately determined by capillary tubing or micropipettes which are introduced within gingival sulcus after adequate isolation and drying. Micropipettes allow precise collection of GCF from a given site in a fixed volume. This technique yields undiluted native GCF, but the collection of adequate samples may take >30 min which may pose risk of trauma to the tissues. Another drawback is the difficulty in removing the complete sample from the tubing [18]. The conclusion in accordance with a published work suggests that standard operating procedure of GCF specimen involves the use of perio-paper as best transporting source through intra-gingival absorption where the strip is kept sub-gingivally for 30 s. The components of the GCF can be analyzed by ELISA which is the most preferred method of analysis [19]. Environment before collection must be sterile and well isolated, and the perio-paper must be held at place for 30 s to avoid contamination with blood or saliva. This approach offers numerous benefits including ease of use, allowing collection from individual sites and a quick technique that avoids tissue trauma.

### 2.6. Conventional Diagnostic Measure Versus GCF

In the gingival sulcus, one surface is tooth while the other is the wall of the pocket, therefore during the event of phase change from transudate to exudate form, the first seen pathology is inflammation of gingival connective tissue, that is clinically signified as gingivitis [20]. Later, when the extension of deterioration involves bone, it is called periodontitis. Periodontitis being one of the most prevalent diseases known to humankind and its severe forms mark 10% of adults. Besides, it’s critical types were recently classified as the sixth most widespread ailment across the globe [21]. Diagnostic potential of the sulcular fluid was known more than six decades ago and since the 1950s this domain is under the discussion of research experts but has failed to achieve a unanimous acceptance to implement it as a clinically significant tool till to date.

Currently, research strategies are emphasizing more on advance and sensitive methods to point active phases and sites of periodontal diseases to differentiate the treatment modality and need as per the requirement [22]. The routine practice uses subjective trends that are based on three aspects. Firstly, the clinical signs and symptoms such as bleeding on probing, change in the gingival color, contour, texture, size plays a crucial role in the establishment of the gingival inflammation, Secondly, determination of periodontal probing depth (PPD) with the help of a periodontal probe. The most commonly used probe is William′s periodontal probe. The other probes used to detect PPD are Community Periodontal Index of Treatment Needs–Clinical probe (CPITN (C)) and Community Periodontal Index of Treatment Needs–Epidemiological probe (CPITN(E)) [23]. Whereas, the later CPITN (E) is commonly used in epidemiological screening. Lastly, the radiological evaluation of alveolar bone loss is essential to ascertain the grade of periodontitis.

Additionally, the present diagnostic method is evaluated based on clinical perspectives such as plaque score (PS), gingival index (GI), clinical attachment loss (CAL), bleeding on probing (BOP), periodontal probing depth (PPD) and radio-graphical analysis. These parameters can only render information about the former destruction of periodontium instead of illustrating the future state of the tissues [11,24]. Thus, it is mandatory to explore a new method of periodontal diagnosis that can provide information about future disease outcomes. The abovementioned subjective criteria is not proficient enough to diagnose the periodontal diseases with reproducibility. Therefore, core objective criteria are colossally essential at present to enable a practitioner to pre-emptively diagnose the condition/stage and intervene the pathophysiology for treatment and management.

GCF nevertheless, is a competent instrument that can influence the advancement of proteomics to the next level. The pathophysiology of periodontal diseases can be gauged by analysis of the protein composition of GCF as various cells and inflammatory biomarkers such as enzymes, cytokines, and local tissue degradation products are discharged in the GCF during the inflammatory state. Extensive use and study of GCF in different perspectives can bridge the gap in understanding many bi-directional diseases (oral-systemic and systemic-oral) and a wide range of oral conditions. In this article, the novelty of GCF is suggested in multiple maladies. Firstly, a wide variety of evidence-based studies have reported that there is no other ideal specimen that is naturally collectable, inexpensive, and biocompatible and with no post complication, which outstands GCF at both practitioner and patient level. It helps reduce the patient’s pre-test anxiety and fear along with a decrease in chair site time. Evidence showed that GCF specimen were found positive for periodontal disease [15,25], where authentic biological mediators which correspond with the pathophysiology of periodontitis in the harmonious pattern were also ascertained. Hence, it will work as a prism to identify any disease with its constituent to cater for easy management of the condition.

### 2.7. GCF as a Biomarker

GCF has found to encompass an extensive range of bio-indicators [26], this property makes it more lucrative for professional use in the crevicular fluid and has shown encouraging co-relation between proteases, collagenase levels and pocket depth derangements among periodontal disease individuals which confirms that clinical parameters can now confidently be explained through this unique fluid [27]. Likewise, a claimable level of protein carbonyl is observed in this fluid that is linked with the initial stage and recovered stages of periodontitis patients [28]. Literature based on an extensive collection of studies through a meta-analysis has expressed realistic long-term benefits of this approach. However, it recommends the use of more than one marker as a standard. Multiple studies have suggested MMP-8 and IL-1β, various others have found that MMP-8 is the most reliable indicator present persistently in periodontitis [29,30]. Another study identified a positive correlation of increased Interleukin-1β (IL-1β) and Interleukin-6 (IL-6) in GCF with severe BOP and increased pocket depth [31].

Several other proteolytic and hydrolytic enzyme biomarkers also provide useful information to predict, diagnose, and monitor periodontal diseases. Alkaline phosphatase (ALP) is a very important indicator of bone formation and its presence in the GCF indicates inflammation and destruction of periodontal tissues. The severity of the periodontal disease is positively correlated to the level of ALP [32]. Lactate dehydrogenase (LDH) activity is elevated with increasing probing depth [33]. Aspartate aminotransferase (AST) is a non-specific marker for cell death and necrosis and its activity is associated with the severity of periodontitis [34]. Finally, cathepsin-B aids in distinguishing periodontitis from gingivitis by serving as a predictor of attachment loss [35].

GCF also contain various bone-related biomarkers to reflect the disease status of periodontal tissues [36]. Osteocalcin (OC) is the most specific biomarker of osteoblast function [37]. An increase in OC level in GCF is associated with high rates of bone turnover and seen during increased periodontal disease activity [38]. Calprotectin alters the immune response by inhibiting the immunoglobulin production and also plays a role in neutrophil recruitment and activation. Higher levels of calprotectin levels are reported in periodontitis patients [38]. Osteopontin (OPN) is mainly produced by osteoblast and macrophages and its raised levels are found associated with periodontal disease. Similarly, osteonectin is an important biomarker associated with the periodontal disease status and its increasing levels correlates with the increase in pocket depth [38,39].

Cell death and tissue breakdown products serve as reliable markers for tissue destruction. Different glycosaminoglycans are found depending on the tissue, chondroitin-4-sulfate is the most promising marker as it reflects bone degradation [12]. Elevated glycosaminoglycan concentration in GCF depicts the active destruction of periodontal tissue [40]. Fibrinogen plays a pivotal role in a variety of cellular activities and is considered an active player in inflammation [41]. It is a marker for periodontal disease status as specific fibrinogen fragments are involved in the pathogenesis of periodontitis [38].

Several other components of GCF are also shown to reflect the periodontal disease status that includes neutrophil elastase [42], periostin [43], Chitinase-3-like protein 1 (also known as YKL-40) [44], lysophosphatidic acid (LPA) [45], human beta-defensins (hBDs) [46], hypoxia-inducible factor-1α (HIF-1α) [47], osteoprotegerin [48], and anti-Hsp-70 (heat shock protein family A) [49]. In light of innumerable proactive studies, it has already been suggested that this is an ideal fluid as a strong and unmatchable latest chemical gizmo that cross matches the clinical parameters too. While negotiating the pros of GCF, there exist very few studies presenting that levels of biomarkers are not significantly different for healthy and diseased individuals [50] which need to produce more clarity in studies that suggest a scientific evolutionary rationale for these indicators and this tool.

**Figure 3:**
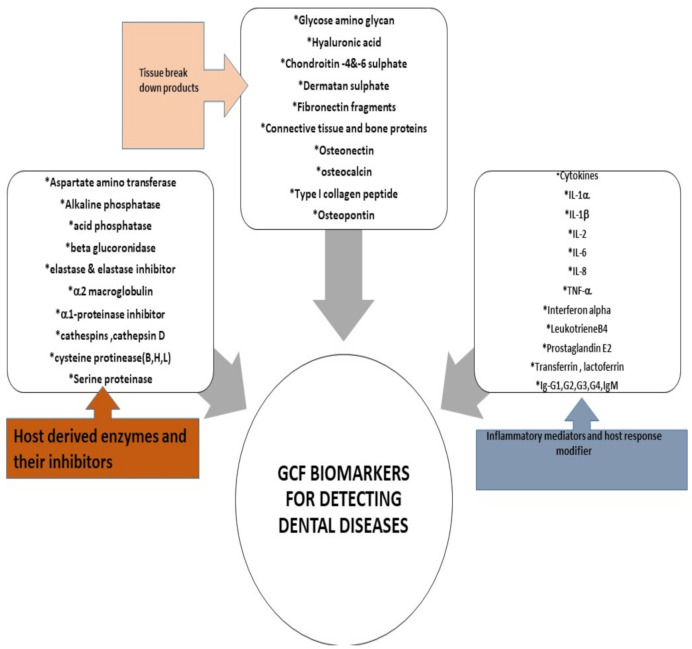
GCF Biomarkers for detecting dental diseases [8,22].

Many well-renowned researchers in the field have put forward the role of numerous proteomes as validated biomarker for the initiation and inhibition of periodontal pathological process [30,51]. In 2016, a definite and scientifically accelerative proof presented by *Barros* et al. team indicated that GCF is a source of multiple biomarkers that potentially indicate disease presence with details of active and passive sites [52]. In 2017, Kaur et al. provided a comprehensive overview of the host defense mediators and their expression in the crevicular fluid around the tooth, these studies explicitly favored the use of GCF as a universal tool [53]. Various methods of manipulating GCF for evaluating gingival related diseases have been proposed such as polymerase chain reaction (PCR), enzyme-linked immunosorbent assay (ELISA) or immunofluorometric assay (IFMA). The application of liquid chromatography-mass spectrometry (LC-MS) combined with label-free quantitative proteomic approaches have led to discovery of 154 proteins of human, viral, bacterial, and fungal origin in 2010, that consequently helped scientists to expedite a detailed investigation of both microbial and host protein biochemistry that will open door for exploration of a variety of treatment modalities [54]. Thus, the emerging quantitative and qualitative proteomics will continue to provide new assessment strategies to further unravel the diagnostic and therapeutic role of GCF (Figure 3).

Furthermore, GCF is exploited to analyze the biochemical parameters to identify the periodontal disease at its early stage before the commencement of the clinical damage [8,55]. The current approaches for assessing periodontal disease include PD and BOP. However, the reliability of these tools is questioned by many studies [56]. Moreover, decrease patient compliance due to associated pain with the probing [57] and higher incidence of negative predictive value [58] highlights the need for a less invasive and efficient modality. Increase in the GCF volume is the initial sign of inflammation which is indicative of subclinical inflammation [59]. Detection of hemoglobin in the GCF has been advocated for early diagnosis as this indicator of bleeding in the periodontal pocket can be detected well in advance of BOP [60]. Matrix metalloproteinase-8 (MMP-8) levels can effectively diagnose gingivitis, which is an early inflammatory prerequisite state to periodontitis. Thus, this approach can effectively be employed to prevent periodontitis [61].

In support of the prospects the GCF’s utility as a reliable method for diagnosis, prevention, intervention and management of oral and systemic maladies, a list of studies is shown in the Table 1. The stated appraisals strongly suggest making the best use of this user-friendly specimen which provides a comprehensive profile of multiple host-derived enzymes, tissue breakdown products, inflammatory mediators and host response modifiers using with a multi-markers approach could provide the best assessment method for disease detection.

## 3. Conclusions

In today’s health- and cost-conscious environment, it is essential to make rational and cost-effective decisions for the prevention and treatment of periodontal disease. This prevention and treatment should be based on accurate diagnosis, reducing causative factors and risk management. A wide range of periodontal diseases exist, requiring various types of treatment. Success lies in the establishment of precise diagnoses to detect the disease type with precision which is best provided with the help of GCF–a complete bioinformatics tool. The purpose of this study was to develop a sense among dental practitioners about the use of gingival crevicular fluid as a fluid biopsy specimen.

The goal of periodontal diagnostic procedures via GCF assessment is to provide useful information to the clinician regarding the present periodontal disease location, and severity [80]. These findings serve as a basis for treatment planning and provide essential data during periodontal maintenance and disease-monitoring phases of treatment. Therefore, via this study we want to endorse Specific, Measurable, Achievable, Realistic and Time-bound (SMART) periodontal diagnosis to broaden the horizon of diagnostic criteria, also to shift the conventional paradigm from subjective to objective methods of assessment.

The future holds promising possibilities for GCF as a periodiagnostic tool that offers a non-invasive, efficient and easy to use approach to sample biomarkers of inflammation and bone resorption in the oral cavity [81] and allow to differentiate the active inflamed sites and predict future tissue destruction and to diagnose early signs of periodontitis. Furthermore, it can also allow monitoring of this condition and prompt discovery of new biomarkers that will aid in the development of new therapeutic approaches via host modulatory drugs for periodontal disease treatment leading to more individualized, targeted treatments for oral health.

The major attraction of GCF as a diagnostic marker is the site-specific nature of the sample. This allows laboratory investigations of GCF constituents to be linked to clinical assessments at the site of sample collection which may offer the basis for patient-specific diagnostic tests for periodontal disease. Moreover, GCF as a diagnostic marker can indicate the presence of a disease process before extensive clinical damage has occurred. Finally, the simplicity of its use along with a level of reliability and low cost favors its use over other modalities.

## Figures and Tables

**Table 1:** Studies showing GCF as biomarkers for periodontal health and disease.

Study Title	Year of Study	Sample	Biomarkers	Objective	Outcome	References
Correlation of levels of interleukin 1β in gingival crevicular fluid to the clinical parameters of chronic periodontitis.	2011	GCF	IL-1β	Correlate IL-1β levels with the clinical parameters of the periodontal disease progression.	IL-1β seems to be a strong correlation between periodontal tissue destruction and IL-1β. Furthermore, IL-1β level could also differentiate between active and inactive periodontal lesions.	[62]
Gingival crevicular fluid and plasma acute phase cytokine levels in different periodontal diseases.	2012	GCF	IL1-β	To investigate gingival crevicular fluid (GCF) and plasma acute-phase cytokines, interleukin-1b (IL-1b), interleukin-6 (IL-6), interleukin11 (IL-11), oncostatin M (OSM), and leukemia inhibitory factor (LIF) levels in patients with different periodontal diseases.	Chronic periodontitis (CP) and generalized aggressive periodontitis (GAgP) groups had significantly higher GCF IL-1b, IL-6, and IL-11 levels when compared with the healthy group.	[63]
Gingival crevicular fluid MMP-8 and -13 and TIMP-1 Levels in patients with rheumatoid arthritis and inflammatory periodontal disease.	2009	GCF	MMP-13 MMP-8	To compare GCF levels of matrix metalloproteinase (MMP)-8 and -13 and tissue inhibitor of MMP (TIMP)-1 in patients with rheumatoid arthritis (RA) and systemically healthy counterparts with inflammatory periodontal disease.	The total amounts of MMP-8 were lower in the healthy control group than in RA-gingivitis, RA-periodontitis, and healthy/periodontitis groups. Patients with RA and gingivitis or periodontitis exhibited levels of MMP-8 and -13 and TIMP-1 that were similar to systemically healthy counterparts.	[64]
Correlations between pentraxin 3 or cytokine levels in gingival crevicular fluid and clinical parameters of chronic periodontitis.	2012	GCF	IL-1β	To determine the levels of IL-1β, IL-6, IL-8, TNF-α, IL-10 and PTX3 in GCF from diseased and healthy sites in patients with chronic periodontitis.	The mean level of PTX3 in diseased sites was approximately 10 times higher than in healthy sites. Additionally, the mean levels of IL-1β, IL-6, IL-8, IL-10 and TNF-α were significantly higher in diseased sites as compared to healthy sites.	[65]
Crevicular fluid biomarkers and periodontal disease progression.	2014	GCF, Saliva	MMP-8, MMP-9, Osteoprotegerin, C-reactive Protein and IL-1β.	Assess the ability of a panel of GCF biomarkers as predictors of periodontal disease progression (PDP).	Except for GCF C-reactive protein, all biomarkers were significantly higher in the PDP group compared to stable patients.	[66]
The effect of adjunctive chlorhexidine mouth rinse on GCF MMP-8 and TIMP-1 levels in gingivitis: a randomized placebo-controlled study.	2014	GCF	TIMP-1	To evaluate the effect of adjunctive chlorhexidine (CHX) mouth rinse on GCF MMP-8 and TIMP-1 levels in plaque-associated gingivitis.	CHX usage had no significant effects on the GCF MMP-8 and TIMP-1 levels in plaque-associated gingivitis. However, daily plaque control increased GCF TIMP-1 levels regardless of CHX usage.	[67]
Gingival crevicular fluid levels of MMP-8, MMP-9, TIMP-2, and MPO decrease after periodontal therapy.	2010	GCF	MMP-8, MMP-9, TIMP-2, and MPO	Comparing the levels of MMP-8, TIMP-1 and TIMP-2, Myeloperoxidase (MPO), and MMP-9 in the GCF of CP patients and controls at baseline and 3 months after non-surgical therapy.	Higher levels of MMP-8, TIMP-2, MPO, and 87 kDa MMP-9 were found in the GCF of patients compared with controls, and these markers decreased 3 months after periodontal therapy.	[68]
Effects of photodynamic therapy on clinical and gingival crevicular fluid inflammatory biomarkers in chronic periodontitis: A split-mouth randomized clinical trial.	2014	GCF	Interleukin-1b, tumor necrosis factor (TNF)-a, and MMP-8 and -9	Compare the clinical parameters and cytokine profiles in the gingival crevicular fluid of patients with moderate-to-severe chronic periodontitis who have been treated using scaling and root planing (SRP) alone or SRP + photodynamic therapy (PDT).	It was concluded that the addition of PDT (using a 638-nm laser and toluidine blue) to SRP did not provide any additional improvements in the clinical or biologic parameters of periodontitis.	[69]
Levels of gingival crevicular metalloproteinases-8 and -9 in periodontitis.	2010	GCF	MMP-8 and MMP-9	To determine the levels of GCF MMP-8 and -9 in patients with periodontitis and healthy controls.	Crevicular MMP-8 and -9 may serve as biomarkers of periodontal disease and aid in early detection of periodontitis.	[70]
Biomarkers of periodontitis in oral fluids.	2008	GCF, saliva	MMP-8, MMP-9, and MMP-2	Compare levels of MMP-2, MMP-9 in GCF and MMP-8 in saliva sample from healthy subjects and patients with gingivitis and periodontitis.	Elevated MMP-8 levels in periodontitis and gingivitis subjects compared to controls, similarly increased MMP-9 levels in GCF of both groups than healthy group. Whereas, MMP-2 was lower in both groups compared to the healthy group.	[71]
Association of gingival crevicular fluid biomarkers during periodontal maintenance with subsequent progressive periodontitis.	2010	GCF	MMP-8, IL-1β, SDD, ICTP (Carboxyterminaltelopeptide cross-link fragment of type I collagen)	Correlate GCF biomarkers of inflammation and bone resorption with subsequent periodontal attachment and bone loss in a longitudinal trial of a matrix metalloproteinase (MMP)-inhibitor.	Elevated GCF biomarkers of inflammation and bone resorption from a small number of moderate/deep sites hold promise in identifying patients vulnerable to progressive periodontitis, and SDD may modify that risk.	[72]
Detection of gingival crevicular fluid MMP-8 levels with different laboratory and chair-side methods.	2010	GCF	MMP-8	To compare four methods for GCF MMP-8 detection; IFMA, dentoAnalyzer, MMP-8 dipstick test, Amersham ELISA kit.	Investigated levels of GCF MMP-8 with two different chair-side and two laboratory methods. IFMA, dentoAnalyzer and MMP-8 specific chair-side dip-stick test results were well in line.	[73]
Characterization of progressive periodontal lesions in chronic periodontitis patients: levels of chemokines, cytokines, matrix metalloproteinase-13, periodontal pathogens and inflammatory cell.	2008	GCF	IL-1β, RANK-L, MCP-1, TNF-α, MMP-13, periodontal pathogen	To determine the levels of chemokine, cytokine, MMP-13, periodontal pathogen, and inflammatory cells in periodontal sites by active periodontal connective destruction.	Higher RANK-L, IL-1β and MMP-13 activity levels observed in active site.	[74]
Gingival crevicular fluid leptin levels in periodontitis patients with long term and heavy smoking.	2006	GCF	Leptin levels	Assess the levels of leptin in patients with chronic periodontitis via GCF.	High GCF levels of leptin in healthy sites in periodontitis patients suggesting a protective role of Leptin in periodontal disease.	[75]
Dentin phosphoprotein in gingival crevicular fluid during root resorption.	2004	GCF	Dentine phosphoprotein (DPP)	External apical root resorption measurement using dentine phosphoprotein during orthodontic treatment.	DPP is most abundant in GCF of an exfoliating tooth, the lesser amount is found in apical root resorption in teeth associated with orthodontic treatment.	[76]
Clinical and biological indicators of dental caries and periodontal disease in adolescents with or without obesity.	2014	GCF samples, Unstimulated and paraffin stimulated whole saliva samples.	SIgAlevels, BMI	This study aimed to assess clinical, microbiological and inflammatory parameters as indicators for caries and periodontal disease.	No significant differences in any of the measured inflammatory markers in GCF were observed between the two groups.	[77]
The concentrations of IL-8 and IL-6 in gingival crevicular fluid during nickel-chromium alloy porcelain crown restoration.	2013	GCF sample	IL-8 and IL-6	Explored gum irritation and cytotoxicity caused by nickel-chromium (Ni-Cr) alloy porcelain by IL-8, IL-6 and GCF volumes at different time point’ speri-crown restoration.	IL-8 and IL-6 during nickel-chromium alloy porcelain crown restoration relates to cytotoxicity induced by Ni–Cr alloy. Thus, should be used with caution in clinical practice, especially in the female population.	[78]
Clinical and microbiological evaluation of marginal gingiva around direct composite veneers.	2016	GCF sample	Clinical and microbiological markers	To evaluate clinically and microbiologically the gingival margins around direct composite veneers.	The volumetric analysis of GCF at the baseline showed lower mean GCF compared to post-veneer placement after 8 weeks, which was statically significant. Microbiologically, no statistically significant difference was seen at the baseline and 8 weeks after veneers placement. Therefore, direct composite veneers can be used as a treatment modality for mild to moderate fluorosis cases.	[79]

## Data Availability

Data will be provided on reasonable request from Deepti Shrivastava, sdeepti20@gmail.com.

## References

[1] Goodson J.M., Tanner A.C.R., Haffajee A.D., Sornberger G.C., Socransky S.S. (1982). Patterns of progression and regression of advanced destructive periodontal disease. J. Clin. Periodontol..

[2] Albandar J.M. (2002). Periodontal diseases in North America. Periodontology 2000.

[3] Tonetti M.S., Jepsen S., Jin L., Otomo-Corgel J. (2017). Impact of the global burden of periodontal diseases on health, nutrition and wellbeing of mankind: A call for global action. J. Clin. Periodontol..

[4] Darveau R.P. (2010). Periodontitis: A polymicrobial disruption of host homeostasis. Nat. Rev. Microbiol..

[5] Ebersole J.L. (2003). Humoral immune responses in gingival crevice fluid: Local and systemic implications. Periodontology 2000.

[6] Zhou J., Yao Y., Jiao K., Zhang J., Zheng X., Wu F., Hu X., Li J., Yu Z., Zhang G. (2017). Relationship between Gingival Crevicular Fluid Microbiota and Cytokine Profile in Periodontal Host Homeostasis. Front. Microbiol..

[7] Pei J., Li F., Xie Y., Liu J., Yu T., Feng X. (2020). Microbial and metabolomic analysis of gingival crevicular fluid in general chronic periodontitis patients: Lessons for a predictive, preventive, and personalized medical approach. EPMA J..

[8] Khurshid Z., Mali M., Naseem M., Najeeb S., Zafar M.S. (2017). Human Gingival Crevicular Fluids (GCF) Proteomics: An Overview. Dent. J..

[9] Griffiths G.S. (2003). Formation, collection and significance of gingival crevice fluid. Periodontology 2000.

[10] Brill N., Krasse B.O. (1958). The Passage of Tissue Fluid into the Clinically Healthy Gingival Pocket. Acta Odontol. Scand..

[11] Armitage G.C. (2004). The complete periodontal examination. Periodontology 2000.

[12] Oswal S., Dwarakanath C. (2010). Relevance of gingival crevice fluid components in assessment of periodontal disease—A critical analysis. J. Indian Soc. Periodontol..

[13] Günday S., Topcu A.O., Ercan E., Yamalik N. (2014). Analysis of Daytime Variations in Gingival Crevicular Fluid: A Circadian Periodicity?. J. Periodontol..

[14] Winer R.A., O’Donnell L.J., Chauncey H.H., McNamara T.F. (1970). Enzyme Activity in Periodontal Disease. J. Periodontol..

[15] Arias-Bujanda N., Regueira-Iglesias A., Alonso-Sampedro M., González-Peteiro M.M., Mira A., Balsa-Castro C., Tomás I. (2018). Cytokine thresholds in gingival crevicular fluid with potential diagnosis of chronic periodontitis differentiating by smoking status. Sci. Rep..

[16] Skapski H., Lehner T. (1976). A crevicular washing method for investigating immune components of crevicular fluid in man. J. Periodontal Res..

[17] Shrivastava D., Srivastava K.C., Dayakara J.K., Sghaireen M.G., Gudipaneni R.K., Al-Johani K., Baig M.N., Khurshid Z. (2020). BactericidalActivity of Crevicular Polymorphonuclear Neutrophils in Chronic Periodontitis Patients and Healthy Subjects under the Influence of Areca Nut Extract: An In Vitro Study. Appl. Sci..

[18] Ghallab N.A. (2018). Diagnostic potential and future directions of biomarkers in gingival crevicular fluid and saliva of periodontal diseases: Review of the current evidence. Arch. Oral Biol..

[19] Gul S.S., Griffiths G.S., Stafford G.P., Al-Zubidi M.I., Rawlinson A., Douglas C.W. (2017). Investigation of a novel predictive biomarker profile for the outcome of periodontal treatment. J. Periodontol..

[20] Nimbulkar G., Garacha V., Shetty V., Bhor K., Srivastava K.C., Shrivastava D., Sghaireen M.G. (2020). Microbiological and clinical evaluation of Neem gel and Chlorhexidine gel on dental plaque and gingivitis in 20–30 years old adults: A randomized parallel-armed, double-blinded controlled trial. J. Pharm. Bioallied Sci..

[21] Larsson L. (2017). Current Concepts of Epigenetics and Its Role in Periodontitis. Curr. Oral Health Rep..

[22] Qasim S.S.B., Al-Otaibi D., Al-Jasser R., Gul S.S., Zafar M.S. (2020). An Evidence-Based Update on the Molecular Mechanisms Underlying Periodontal Diseases. Int. J. Mol. Sci..

[23] Nomura Y., Okada A., Kakuta E., Gunji T., Kajiura S., Hanada N. (2016). A new screening method for periodontitis: An alternative to the community periodontal index. BMC Oral Health.

[24] Lindhe J., Haffajee A.D., Socransky S.S. (1983). Progression of periodontal disease in adult subjects in the absence of periodontal therapy. J. Clin. Periodontol..

[25] Buduneli N. (2019). Biomarkers in periodontal health and disease: Rationale, benefits, and future directions. Biomarkers in Periodontal Health and Disease: Rationale, Benefits, and Future Directions.

[26] Subrahmanyam M.V., Sangeetha M. (2003). Gingival crevicular fluid a marker of the periodontal disease activity. Indian J. Clin. Biochem..

[27] De Alencar Silva F.G., Gomes S.C. (2009). Validation of an alternative absorbent paper for collecting gingival crevicular fluid/Validação de um papel absorvente para coleta de Fluido Crevicular Gengival. Periodontia.

[28] Konopka Ł., Pietrzak A., Brzezińska-Błaszczyk E. (2012). Effect of scaling and root planing on interleukin-1β, interleukin-8 and MMP-8 levels in gingival crevicular fluid from chronic periodontitis patients. J. Periodontal Res..

[29] Majeed Z.N., Philip K., Alabsi A.M., Pushparajan S., Swaminathan D. (2016). Identification of Gingival Crevicular Fluid Sampling, Analytical Methods, and Oral Biomarkers for the Diagnosis and Monitoring of Periodontal Diseases: A Systematic Review. Dis. Markers.

[30] Graves D. (2008). Cytokines that Promote Periodontal Tissue Destruction. J. Periodontol..

[31] Offenbacher S., Barros S.P., Singer R.E., Moss K., Williams R.C., Beck J.D. (2007). Periodontal disease at the biofilm-gingival interface. J. Periodontol..

[32] Júnior A.A.B., Pallos D., Cortelli J.R., Saraceni C.H.C., Queiroz C.S. (2010). Evaluation of organic and inorganic compounds in the saliva of patients with chronic periodontal disease. Rev. Odonto Ciência.

[33] Wong D.T. (2006). Salivary diagnostics powered by nanotechnologies, proteomics and genomics. J. Am. Dent. Assoc..

[34] Oringer R.J., Howell T.H., Nevins M.L., Reasner D.S., Davis G.H., Sekler J., Fiorellini J.P. (2001). Relationship Between Crevicular Aspartate Aminotransferase Levels and Periodontal Disease Progression. J. Periodontol..

[35] Eley B.M., Cox S.W. (1996). The relationship between gingival crevicular fluid cathepsin B activity and periodontal attachment loss in chronic periodontitis patients: A 2-year longitudinal study. J. Periodontal Res..

[36] Ahmad P., Arshad A.I., Bella E.D., Khurshid Z., Stoddart M. (2020). Systemic Manifestations of the Periodontal Disease: A Bibliometric Review. Molecules.

[37] Fassbender W.J., Steinhauer B., Stracke H., Schumm-Draeger P.-M., Usadel K.-H. (2002). Validation of a new automated immunoassay for measurement of intact osteocalcin. Clin. Lab..

[38] Khiste S.V., Ranganath V., Nichani A.S., Rajani V. (2011). Critical analysis of biomarkers in the current periodontal practice. J. Indian Soc. Periodontol..

[39] Bowers M.R., Fisher L.W., Termine J.D., Somerman M.J. (1989). Connective tissue-associated proteins in crevicular fluid: Potential markers for periodontal diseases. J. Periodontol..

[40] Airila-Månsson S., Söder B., Kari K., Meurman J.H. (2006). Influence of Combinations of Bacteria on the Levels of Prostaglandin E2, Interleukin-1β, and Granulocyte Elastase in Gingival Crevicular Fluid and on the Severity of Periodontal Disease. J. Periodontol..

[41] Huynh Q.N., Wang S., Tafolla E., Gansky S.A., Kapila S., Armitage G.C., Kapila Y.L. (2002). Specific Fibronectin Fragments as Markers of Periodontal Disease Status. J. Periodontol..

[42] Aral C.A., Ölçer S.N., Aral K., Kapila Y. (2020). Oxidative stress, neutrophil elastase and IGFBP7 levels in patients with oropharyngeal cancer and chronic periodontitis. Oral Dis..

[43] Sophia K., Suresh S., Sudhakar U., Cader S.A., Vardhini V.M., Arunachalam T.L., Jean S.C. (2020). Comparative Evaluation of Serum and Gingival Crevicular Fluid Periostin Levels in Periodontal Health and Disease: A Biochemical Study. Cureus.

[44] Kumar P.A., Kripal K., Chandrasekaran K., Bhavanam S.R. (2019). Estimation of YKL-40 Levels in Serum and Gingival Crevicular Fluid in Chronic Periodontitis and Type 2 Diabetes Patients among South Indian Population: A Clinical Study. Contemporary Clin. Dent..

[45] Hashimura S., Kido J., Matsuda R., Yokota M., Matsui H., Inoue-Fujiwara M., Inagaki Y., Hidaka M., Tanaka T., Tsutsumi T. (2020). A low level of lysophosphatidic acid in human gingival crevicular fluid from patients with periodontitis due to high soluble lysophospholipase activity: Its potential protective role on alveolar bone loss by periodontitis. Biochim. Biophys. Acta Mol. Cell Biol. Lipids.

[46] Pereira A.G., Costa L.C.M., Soldati K.R., De Abreu M.H.N.G., Costa F.O., Zandim-Barcelos D.L., Cota L.O.M. (2020). Gingival Crevicular Fluid Levels of Human Beta-defensin 2 and 3 in Healthy and Diseased Sites of Individuals with and without Periodontitis. J. Int. Acad. Periodontol..

[47] Zorina O.A., Amkhadova M.A., Abaev Z.M., Khamukova A.A., Demidova A.A. (2020). Hypoxia-dependent transcriptional control of activity of destructive inflammatory and malignant periodontium changes. Stomatologiia.

[48] Kinney J.S., Morelli T., Oh M., Braun T.M., Ramseier C.A., Sugai J.V., Giannobile W.V. (2014). Crevicular fluid biomarkers and periodontal disease progression. J. Clin. Periodontol..

[49] Takai H., Furuse N., Ogata Y. (2020). Anti-heat shock protein 70 levels in gingival crevicular fluid of Japanese patients with chronic periodontitis. J. Oral Sci..

[50] Najeeb S., Zafar M.S., Khurshid Z., Zohaib S., Almas K. (2016). The Role of Nutrition in Periodontal Health: An Update. Nutrients.

[51] Garlet G.P. (2010). Critical reviews in oral biology & medicine: Destructive and protective roles of cytokines in periodontitis: A re-appraisal from host defense and tissue destruction viewpoints. J. Dent. Res..

[52] Barros S.P., Williams R.C., Offenbacher S., Morelli T. (2016). Gingival crevicular fluid as a source of biomarkers for periodontitis. Periodontology 2000.

[53] Kaur G., Mohindra K., Singla S. (2017). Autoimmunity—Basics and link with periodontal disease. Autoimmun. Rev..

[54] Bostanci N., Bao K., Greenwood D., Silbereisen A., Belibasakis G.N. (2019). Periodontal disease: From the lenses of light microscopy to the specs of proteomics and next-generation sequencing. Advances in Clinical Chemistry.

[55] Khurshid Z., Warsi I., Moin S.F., Slowey P.D., Latif M., Zohaib S., Zafar M.S. (2021). Biochemical analysis of oral fluids for disease detection. Advances in Clinical Chemistry.

[56] Armitage G.C., Svanberc G.K., Loe H. (1977). Microscopic evaluation of clinical measurements of connective tissue attachment levels. J. Clin. Periodontol..

[57] Heft M.W., Perelmuter S.H., Cooper B.Y., Magnusson I., Clark W.B. (1991). Relationship between gingival inflammation and painfulness of periodontal probing. J. Clin. Periodontol..

[58] Lang N.P., Adler R., Joss A., Nyman S. (1990). Absence of bleeding on probing an indicator of periodontal stability. J. Clin. Periodontol..

[59] Rüdin H.J., Overdiek H.F., Rateitschak K.H. (1970). Correlation between sulcus fluid rate and clinical and histological inflammation of the marginal gingiva. Helv. Odontol. Acta.

[60] Ito H., Numabe Y., Hashimoto S., Sekino S., Murakashi E., Ishiguro H., Sasaki D., Yaegashi T., Takai H., Mezawa M. (2016). Correlation between Gingival Crevicular Fluid Hemoglobin Content and Periodontal Clinical Parameters. J. Periodontol..

[61] Hong I., Pae H.-C., Song Y.W., Cha J.-K., Lee J.-S., Paik J.-W., Choi S.-H. (2020). Oral Fluid Biomarkers for Diagnosing Gingivitis in Human: A Cross-Sectional Study. J. Clin. Med..

[62] Chaudhari A.U., Byakod G.N., Waghmare P.F., Karhadkar V.M. (2011). Correlation of Levels of Interleukin-1β in Gingival Crevicular Fluid to the Clinical Parameters of Chronic Periodontitis. J. Contemp. Dent. Pr..

[63] Becerik S., Öztürk V.Ö., Atmaca H., Atilla G., Emingil G. (2012). Gingival Crevicular Fluid and Plasma Acute-Phase Cytokine Levels in Different Periodontal Diseases. J. Periodontol..

[64] Bıyıkoğlu B., Buduneli N., Kardeşler L., Aksu K., Pitkala M., Sorsa T. (2009). Gingival Crevicular Fluid MMP-8 and −13 and TIMP-1 Levels in Patients with Rheumatoid Arthritis and Inflammatory Periodontal Disease. J. Periodontol..

[65] Fujita Y., Ito H., Sekino S., Numabe Y. (2012). Correlations between pentraxin 3 or cytokine levels in gingival crevicular fluid and clinical parameters of chronic periodontitis. Odontology.

[66] Wassall R.R., Preshaw P.M. (2016). Clinical and technical considerations in the analysis of gingival crevicular fluid. Periodontology 2000.

[67] Türkoğlu O., Becerik S., Tervahartiala T., Sorsa T., Atilla G., Emingil G. (2014). The effect of adjunctive chlorhexidine mouthrinse on GCF MMP-8 and TIMP-1 levels in gingivitis: A randomized placebo-controlled study. BMC Oral Health.

[68] Marcaccini A.M., Meschiari C.A., Zuardi L.R., de Sousa T.S., Taba M., Teofilo J.M., Jacob-Ferreira A.L.B., Tanus-Santos J.E., Novaes A.B., Gerlach R.F. (2010). Gingival crevicular fluid levels of MMP-8, MMP-9, TIMP-2, and MPO decrease after periodontal therapy. J. Clin. Periodontol..

[69] Pourabbas R., Kashefimehr A., Rahmanpour N., Babaloo Z., Kishen A., Tenenbaum H.C., Azarpazhooh A. (2014). Effects of Photodynamic Therapy on Clinical and Gingival Crevicular Fluid Inflammatory Biomarkers in Chronic Periodontitis: A Split-Mouth Randomized Clinical Trial. J. Periodontol..

[70] Rai B., Kaur J., Jain R., Anand S.C. (2010). Levels of gingival crevicular metalloproteinases-8 and -9 in periodontitis. Saudi Dent. J..

[71] Rai B., Kharb S., Jain R., Anand S.C. (2008). Biomarkers of periodontitis in oral fluids. J. Oral Sci..

[72] Reinhardt R.A., Stoner J.A., Golub L.M., Lee H.-M., Nummikoski P.V., Sorsa T., Payne J.B. (2010). Association of Gingival Crevicular Fluid Biomarkers During Periodontal Maintenance with Subsequent Progressive Periodontitis. J. Periodontol..

[73] Sorsa T., Hernández M., Leppilahti J., Munjal S., Netuschil L., Mäntylä P. (2010). Detection of gingival crevicular fluid MMP-8 levels with different laboratory and chair-side methods. Oral Dis..

[74] Silva N., Dutzan N., Hernández M., Dezerega A., Rivera O., Aguillón J.C., Aravena O., Lastres P., Pozo P., Vernal R. (2008). Characterization of progressive periodontal lesions in chronic periodontitis patients: Levels of chemokines, cytokines, matrix metalloproteinase-13, periodontal pathogens and inflammatory cells. J. Clin. Periodontol..

[75] Bozkurt F.Y., Ay Z.Y., Sütçü R., Delibas N., Demirel R. (2006). Gingival Crevicular Fluid Leptin Levels in Periodontitis Patients with Long-Term and Heavy Smoking. J. Periodontol..

[76] Mah J., Prasad N. (2004). Dentine phosphoproteins in gingival crevicular fluid during root resorption. Eur. J. Orthod..

[77] Fadel H.T., Pliaki A., Gronowitz E., Mårild S., Ramberg P., Dahlén G., Yucel-Lindberg T., Heijl L., Birkhed D. (2014). Clinical and biological indicators of dental caries and periodontal disease in adolescents with or without obesity. Clin. Oral Investig..

[78] Yu L., Su J., Zou D., Mariano Z. (2013). The concentrations of IL-8 and IL-6 in gingival crevicular fluid during nickel–chromium alloy porcelain crown restoration. J. Mater. Sci. Mater. Med..

[79] Tiwari I., Mannava P., Shetty S., Singh A., Shrivastava L., Verma S. (2016). Clinical and Microbiological Evaluation of Marginal Gingiva Around Direct Composite Veneers. J. Int. Oral Health.

[80] Hamid H., Khurshid Z., Adanir N., Zafar M.S., Zohaib S. (2020). COVID-19 Pandemic and Role of Human Saliva as a Testing Biofluid in Point-of-Care Technology. Eur. J. Dent..

[81] Sanikop S., Patil S., Agrawal P. (2012). Gingival crevicular fluid alkaline phosphatase as a potential diagnostic marker of periodontal disease. J. Indian Soc. Periodontol..

